# Synergistic bioconversion of organic waste by black soldier fly (*Hermetia illucens*) larvae and thermophilic cellulose-degrading bacteria

**DOI:** 10.3389/fmicb.2023.1288227

**Published:** 2024-01-10

**Authors:** Mingying Shao, Xiao Zhao, Kashif Ur Rehman, Minmin Cai, Longyu Zheng, Feng Huang, Jibin Zhang

**Affiliations:** ^1^Institute of Tropical Agricultural Technology, Hainan Vocational University, Haikou, Hainan, China; ^2^National Key Laboratory of Agricultural Microbiology, National Engineering Research Center of Microbial Pesticides, College of Life Science and Technology, Huazhong Agricultural University, Wuhan, China; ^3^Hubei Hongshan Laboratory, Wuhan, China; ^4^Department of Microbiology, Faculty of Veterinary and Animal Sciences, The Islamia University of Bahawalpur, Bahawalpur, Pakistan; ^5^German Institute of Food Technologies, Quakenbrück, Germany

**Keywords:** BSFL, Wuzhishan pig, gut microbe, manure, fiber degradation, bioconversion

## Abstract

**Introduction:**

This study examines the optimum conversion of Wuzhishan pig manure by Black Soldier Fly Larvae (BSFL) at various phases of development, as well as the impact of gut microbiota on conversion efficiency.

**Method and results:**

In terms of conversion efficiency, BSFL outperformed the growing pig stage (GP) group, with significantly higher survival rates (96.75%), fresh weight (0.23 g), and larval conversion rate (19.96%) compared to the other groups. Notably, the GP group showed significant dry matter reductions (43.27%) and improved feed conversion rates (2.17). Nutritional composition varied, with the GP group having a lower organic carbon content. High throughput 16S rRNA sequencing revealed unique profiles, with the GP group exhibiting an excess of *Lactobacillus* and *Clostridium*. Promising cellulose-degrading bacteria in pig manure and BSFL intestines, including *Bacillus cereus* and *Bacillus subtilis*, showed superior cellulose degradation capabilities. The synergy of these thermophilic bacteria with BSFL greatly increased conversion efficiency. The BSFL1-10 group demonstrated high growth and conversion efficiency under specific conditions, with remarkable larval moisture content (71.11%), residual moisture content (63.20%), and waste reduction rate (42.28%).

**Discussion:**

This study sheds light on the optimal stages for BSFL conversion of pig manure, gut microbiota dynamics, promising thermophilic cellulose-degrading bacteria, and the significant enhancement of efficiency through synergistic interactions. These findings hold great potential for sustainable waste management and efficient biomass conversion, contributing to environmental preservation and resource recovery.

## 1 Introduction

The effective handling of agricultural waste, particularly pig manure, is a crucial concern for modern agricultural operations. The exponential rise of large-scale pig farming, in particular, has resulted in increased pig manure, escalating agricultural pollution (De Vrieze et al., 2019). In addition to being nutrient-rich, pig manure also includes a sizable quantity of cellulose, a complex carbohydrate that is essential for the structural integrity of plant cell walls (Shao et al., 2021). When correctly handled, cellulose may be a beneficial energy source and add to soil organic matter, but it also poses environmental risks when it is present in pig manure (Shao et al., 2021). The effective breakdown of pig manure is a challenging undertaking due to the higher resistance to degradation of cellulose-rich waste (Qian et al., 2023). Inadequate management can cause a substance to decompose slowly, releasing greenhouse gasses into the environment including methane and carbon dioxide that fuel climate change (Rehman et al., 2023). In addition, insufficient cellulose breakdown can lead to the buildup of solid debris, which can clog up streams and raise the risk of nutrient runoff and consequent water contamination. Innovative methods to encourage effective decomposition and nutrient recycling are needed to address the environmental issues posed by cellulose content in pig manure and lessen the overall ecological impact of its disposal. Addressing the environmental issues connected with cellulose concentration in pig manure necessitates novel ways that enable effective breakdown and nutrient recycling while minimizing the overall ecological impact of its disposal.

Pineapple crown buds show an unusual convergence of an organic byproduct and an environmental hazard (Idayanti et al., 2022). While crown buds are sometimes seen as waste in the pineapple business, they contain high cellulose content and represent a rich resource for prospective use (Chen et al., 2019). However, if not properly handled, their disposal might cause environmental difficulties (Avila et al., 2022). The buildup of pineapple crown buds leads to agricultural waste, which may lead to increased pollution and resource inefficiency (Idayanti et al., 2022). Recognizing pineapple crown buds as both an underutilized organic resource and a possible environmental hazard highlights the need for new solutions to maximize their worth while minimizing their negative environmental effect.

The Black Soldier Fly Larvae (BSFL), scientifically known as *Hermetia illucens*, appears as a viable answer in this context. BSFL, which is native to tropical and subtropical places across the world, has gained popularity as a resource insect for organic waste treatment (Liu et al., 2008; Zhang et al., 2021). BSFL successfully transform different organic wastes, such as food waste, discarded fruits, and animal manure, into useful nutrients (protein, fat, chitin, melanin) by using their extensive digestive capabilities and diverse nutritional preferences (Wang et al., 2017; Rehman et al., 2017a,b; Mazza et al., 2020). The use of BSFL in animal manure treatment has resulted in significant decreases in manure accumulation rates while increasing larvae biomass (protein and fat) and resulted in substantial reductions in volatile organic compound emissions and harmful microorganism levels (Zhou et al., 2013; Xiao et al., 2018; Chen et al., 2019), effectively mitigating odor issues and the proliferation of houseflies (Beskin et al., 2018).

Moreover, the use of microbe assisted BSF technology has great promise for revolutionizing organic waste management practices (Rehman et al., 2019, 2023; Somroo et al., 2019; Mazza et al., 2020). This new approach to organic waste treatment uses the symbiotic link between BSF larvae and beneficial bacteria to provide an economical and sustainable solution (Rehman et al., 2023). The inherent preference of BSF larvae for ingesting a wide range of organic materials, along with the action of microorganisms in their gut, accelerates the breakdown process (Xiao et al., 2018; Yu et al., 2022). These microorganisms are critical in the breakdown of complex organic substances into simpler ones, effectively transforming waste into useful resources (Yu et al., 2022) This technique produces biomass made of nutrient-rich larvae, organic fertilizers, and bio-based goods in addition to reducing the amount of organic waste produced (Rehman et al., 2017b; Chen et al., 2019). Furthermore, synergy between intestinal microflora and organic waste bacteria has the potential to enhance co-conversion processes, resulting in increased manure utilization (De Smet et al., 2018). Several bacterial strains, including *Kocuria*, *Proteus*, *Bacillus subtilis*, and *Lactobacillus*, have been shown to improve material reduction rates during BSFL-assisted waste treatment (Kariuki et al., 2023). The microbial makeup of BSFL intestines, which is dominated by *Bacteroidetes*, *Proteobacteria*, and *Firmicutes*, contributes considerably to amino acid synthesis and nutrition absorption, impacting larval protein buildup (De Smet et al., 2018; Kariuki et al., 2023).

Additionally, these bacteria can synthesize various enzymes such as α-amylase, protease, lipase, and cellulase, which can work in synergy with the digestive enzymes inside the gut of BSFL (Xiao et al., 2018). Before renewable lignocellulosic residues converted into value-added bio-products through bioconversion, several pretreatment methods are often used to break down their resistant structure, such as dilute acid, steam explosion, and hydrothermal (Agrawal et al., 2021a; Kaur et al., 2023). The efficient hydrolysis of lignocellulosic biomass requires a variety of enzymes, studies have shown that accessory enzymes play a synergistic and critical role in enzymatic hydrolysis of ligno-cellulosic biomass (Agrawal et al., 2018; agrawal et al., 2022). Therefore, adopting BSF microbe-assisted technology for waste management offers a comprehensive and ecologically responsible strategy that addresses trash reduction as well as the production of useful byproducts that support a more sustainable and circular economy.

Therefore, a potential approach hypothesized to handling cellulose-rich Wuzhishan pig manure and the organic waste of pineapple crown buds is the synergistic use of high-temperature cellulose-degrading bacterial strains in conjunction with co-cultivated BSFL. Due to its lower rate of breakdown, cellulose, a crucial component of both pig manure and pineapple crown buds, presents difficulties (Zhang et al., 2021; Idayanti et al., 2022). The resistant cellulose structures can be effectively broken down by using the enzymatic abilities of cellulose-degrading bacteria, especially those that are acclimated to high temperatures (Mazza et al., 2020). The process is further improved by including the voracious eating habits of BSFL, whose diverse food and effective digestive systems complement bacterial enzymatic activity (Rehman et al., 2019, 2023). This collaborative strategy not only speeds up the decomposition of cellulose but also produces beneficial byproducts including biomass from nutrient-rich larvae and converted organic waste into biofertilizer that may be used to enhance soil fertility (Somroo et al., 2019). A sustainable approach to handling waste that is high in cellulose, reducing environmental risks, and establishing a circular economy in the agriculture industry is to embrace these biological synergies (Rehman et al., 2017b). Therefore, the possibility of using BSFL to improve the conversion efficiency of fresh pig manure and pineapple bud crowns is explored in this study. We seek to understand the fundamental processes behind effective pig manure conversion via larval intervention by examining microbial community dynamics and metabolic changes. In addition to laying a technological foundation, this project also offers a practical framework for addressing the complicated biological treatment and recycling issues related to pig breeding waste.

## 2 Materials and methods

### 2.1 Experimental animals and sample collection

In order to conduct this investigation, Wuzhishan pig manure was obtained from the Qiongzhong, Hainan-based Hainan Nongxiang Animal Husbandry Ecological Agriculture Development Co., LTD. At Hainan University, pineapple crown buds were collected from vendors on the night market pedestrian street. During the fattening phase, Wuzhishan pig manure was used as the experiment’s substrate. Pre-weaning pigs (PW, 2.5 to 3.5 kg), post-weaning piglets (WP, 3.5 to 4.5 kg), growing pigs (GP, 7 to 10 kg), and sows (SP, 20 to 26 kg) were all included. The artificial feed (20% cornmeal, 30% alfalfa meal, 50% bran) was used for the rearing of larvae in control (Con) treatment to compare the life history parameters with other treatments during the investigaion The genetically stable *Hermetia illucens* population (Strain Wuhan) developed in 2008 by the State Key Laboratory of Agricultural Microbiology at Huazhong Agricultural University served as the basis for the experimental population employed in the study (Zhou et al., 2013). Each treatment group was inoculated with 400 well-grown larvae of the same size at 6 days of age, with 3 parallel replicate in each group. At the beginning of conversion, the material thickness should be controlled at about 8 cm. The initial water content of the substrate (pig manure and pineapple crown buds) during conversion is about 70%, the room temperature is 28 ± 2°C, and the air humidity is 60–70%. Each fresh sample was collected randomly from each pen and stored in a 2 mL centrifuge tube and kept on ice during transportation. All samples were stored in a − 80°C freezer for cryopreservation until DNA extraction.

### 2.2 DNA extraction and PCR amplification

The physicochemical properties of the transformation of pig manure and waste in Wuzhishan were studied. About 400 well-grown 6-day-old BSFL of the same size were added to each conversion group and sealed with 8 layers of gauze. All experiments were conducted in a greenhouse with a temperature of 28 ± 2°C and a relative humidity of 60–70%. The manure was stirred every morning and evening until the larvae of the Wuhan strain began to emerge as pre-pupae. The collected larvae were then dissected on the day of sampling to collect internal organs. Fecal samples were quickly frozen in liquid nitrogen and stored at −80°C to screen for the microbiome. The 0.2 g sample was accurately weighed, and the total DNA was extracted by FastDNA Kit for Soil (MP Biomedicals, USA) following the manufacturer’s standard protocol. The DNA was extracted using the Thermo Nanodrop 2000 ultramicroscopic spectrophotometer (Thermo Scientific, USA) to detect DNA concentration and purity. The V3-V4 variables region of the bacterial 16S rRNA gene was PCR amplifed using the forward primer 338F (5′- ACTCCTACGGGAGGCAGCA −3′) and reverse primer 806R (5′- GGACTACHVGGGTWTCTAAT −3′). The total reaction volume was 30 μL, and PCR products were purified using the GeneJET gel extraction Kit (Termo Fisher Scientifc, Waltham, MA, USA) according to the manufacturer’s protocol. Illumina NovaSeq 6,000 platform was used for sequencing (pairing end was 2 × 250).

### 2.3 Quantification of target genes

Sequencing was conducted by Biomarker Technologies Corporation (Beijing, China), yielding initial data that required preprocessing to enhance data quality. The following series of data preprocessing steps was applied: First, Trimmomatic v0.33 software was used to filter Raw Reads by removing low-quality sequences and adapter remnants. Subsequently, Cutadapt 1.9.1 software was employed to identify and eliminate primer sequences, resulting in clean reads without any primer sequences (Bolger et al., 2014). The denoising step involved the use of the DADA2 method in QIIME2 2020.6, which integrated paired-end sequences and removed mosaic sequences, yielding a refined dataset free from chimeric reads. To ensure data completeness and quality, FLASH v1.2.7 software was used for high-quality sequence overlap, facilitating the creation of a unified dataset with overlapping sequences (clean reads) (Magoč and Salzberg, 2011). Finally, chimeric sequences were identified and removed using UCHIME v4.2 software to obtain the final dataset of effective reads, free from chimeric sequences and potential artifacts. These data preprocessing steps improved data quality and ensured the integrity of the dataset for downstream analysis (Edgar et al., 2011).

OTUs clustering and species classification analysis were conducted based on valid data. To assign taxonomy to the clustered reads, reads were aligned to the SILVA database. According to the OTUs clustering results, the representative sequences of each OTU was annotated to obtain the corresponding species information and the abundance distribution based on species. In order to study the phylogenetic relationships of different OTUs and the differences of dominant species in different samples, U-search software was used to cluster all the valid data of all samples, and the obtained high-quality sequences were merged, and OTU divided according to 97% sequence similarity. Species annotation and analysis were conducted on the representative OTUS sequences to obtain taxonomic information (Edgar, 2013).

### 2.4 Diversity analysis and functional predictions

Shannon index, Simpson index and Coverage of library were calculated based on QIIME. Using R software, PCA analysis was performed on the community composition structure at the genus level, and the natural distribution characteristics among samples were described by two-dimensional images (Caporaso et al., 2010; Lozupone et al., 2011; Segata et al., 2011). The LC-QTOF platform was used for qualitative and quantitative analysis of metabolomics of the samples (Garcia and Barbas, 2011). PICRUSt2 software was used to annotate the species between the feature sequences to be predicted and the existing phylogenetic trees in the software, and IMG microbial genome data is used to output functional information and then speculate the composition of functional genes in the samples, so as to analyze the functional differences between different samples or groups (Segata et al., 2011).

### 2.5 Screening of thermophilic cellulose-degrading bacteria

The GP group’s Wuzhishan pigs’ waste was collected and sampled. Using a thermometer, the temperature of the waste accumulation was measured in several locations, paying particular attention to regions that had temperatures around 55°C. The quartering method was used to obtain around 500 g of the combination after samples from these hot regions were combined. Following that, these samples were put in sampling bags and kept at 4°C. We dissected Black Soldier Fly (BSF) Wuhan strain larvae that consumed converted Wuzhishan pig manure. The posterior intestine segments’ contents were taken out, put in sample bags, and kept at 4°C for storage. All stages were carried out in a sterile setting and were carried out in accordance with the enrichment process described in the approach (Halimi and Mirsalehian, 2016). In a 1:10 (V/V) ratio, the obtained samples were added to the base medium, well mixed, and homogenized. The resultant culture media was put in a dry oven that was preheated to 55°C, in a room that was completely dark, and allowed to culture for 30 days. The Hutchinson liquid medium was continuously introduced during this time under sterile circumstances. The cellulose degradation circle technique was used to select plates that showed noteworthy effects, and these plates were subsequently chilled to 4°C for further separation and purification. Single colonies with different colony morphology and obvious degradation circle were selected from the preservation plate for stripe culture, which was continued until four generations later, the colonies with the same morphology were made and simply stained, and the morphology of bacteria was observed under an oil immersion and the morphology of bacteria cells was observed under multiple visual fields. The strains with the same morphology were cultured on the inclined side of the basic medium until abundant, and the strains were stored in the refrigerator at 4°C for later use.

### 2.6 Cellulose hydrolysis assessment

Cellulose hydrolysis assessment was performed as previously described (Agrawal et al., 2023). Isolated and purified bacterial strains were inoculated onto cellulose-Congo red agar medium, with 3 spots per dish. After 48 h of cultivation, the colony diameter (d) and the diameter of the transparent zone (D) produced by each bacterial strain were measured. The Up value was calculated using the formula Up = (D/d)^2, which indicated the cellulose degradation ability of the strains.

### 2.7 Enzyme activity assay

Enzyme activity assay was performed as previously described (Agrawal et al., 2017). The isolated cellulose-degrading bacteria were inoculated into 100 mL of LB medium. Samples were taken every 24 h. The collected culture broth was centrifuged at 4°C and 8,000 rpm for 10 min, and the supernatant was used as crude enzyme solution. 0.5 mL of the crude enzyme solution was mixed with 1.5 mL of 0.5% carboxymethyl cellulose sodium solution (0.5 g carboxymethyl cellulose sodium dissolved in 100 mL of citrate buffer) and incubated at 50°C for 30 min. Then, 2 mL of DNS solution was added, followed by boiling in a water bath for 5 min. After cooling and diluting to 10 mL, the absorbance was measured at 540 nm wavelength to calculate enzyme activity.

### 2.8 Filter paper degradation experiment

Filter paper degradation experiment was performed as previously described (Agrawal et al., 2021b). The 5 mL of high-temperature cellulose-degrading bacterial seed solution activated on a basal agar plate at 80°C for 36–48 h was transferred aseptically to 100 mL of sterilized Hutchinson medium in a sterile environment. The mixture was sealed with aluminum foil and cultured at 80°C for 14 days. The filter paper strips were repeatedly washed with a mixture of diluted hydrochloric acid and diluted nitric acid, followed by deionized water. The washed filter paper strips were dried at 80°C until a constant weight was achieved, and the degradation rate of the filter paper was calculated.

### 2.9 Strain identification

The 16S rDNA identification of the strains was performed. Genomic DNA from the strains was prepared for PCR amplification, followed by gel electrophoresis, gel analysis, sequence determination, and sequencing analysis. Based on the amplified primer sequence 27F: 5’-AGAGTTTGATCCTGGCTCAG-3′ and 1492R: 5’-TACGGCTACCTTGTTACGACTT-3′, a Blast comparison was conducted on the National Center for Biotechnology Information (NCBI) GenBank to determine the genus of the strains. The 16S rRNA data for strain identification were uploaded to the NCBI with serial number OR539918-OR539919.

### 2.10 Synergistic conversion experiment between high-temperature cellulose-degrading bacteria and BSFL

Two selected high-temperature cellulose-degrading bacterial strains were co-cultivated with BSFL (Wuhan strain). A two-factor cross experiment was designed: the first factor was the different ratios of Wuzhishan pig feces and pineapple waste materials, and the second factor was the different concentrations of bacterial agents, each with four gradients. The total amount of materials was 1,000 g, with 3 replicates per experimental group. After 3 days of pre-treatment with bacterial agents, 1,000 BSFL (Wuhan strain) were inoculated for a 13-day conversion experiment. The materials were turned over once every 2 days, and samples were collected at fixed time points every 2 days. All samples were stored in a − 80°C ultra-low temperature freezer within 2 h. The material-to-bacterial agent ratios are shown in Table 1.

**Table 1:** Experimental design of microbe assisted black soldier fly larvae with description of total substrate quantity, co-conversion mixture ratio (pig manure and pineapple), and CFU/mL of bacterial strains inoculation.

Treatments	Total substrate weight (g)	Pig manure: pineapple waste	BM strain (%)	DF strain (%)
BSFL_1-12_	1,000	800:200	12%, 10^8^ CFU/mL	24%, 10^8^ CFU/mL
BSFL_1-10_	1,000	800:200	10%, 10^8^ CFU/mL	20%, 10^8^ CFU/mL
BSFL_1-8_	1,000	800:200	8%, 10^8^ CFU/mL	16%, 10^8^ CFU/mL
BSFL_1-6_	1,000	800:200	6%, 10^8^ CFU/mL	12%, 10^8^ CFU/mL
BSFL_2-12_	1,000	600:400	12%, 10^8^ CFU/mL	24%, 10^8^ CFU/mL
BSFL_2-10_	1,000	600:400	10%, 10^8^ CFU/mL	20%, 10^8^ CFU/mL
BSFL_2-8_	1,000	600:400	8%, 10^8^ CFU/mL	16%, 10^8^ CFU/mL
BSFL_2-6_	1,000	600:400	6%, 10^8^ CFU/mL	12%, 10^8^ CFU/mL
BSFL_3-12_	1,000	400:600	12%, 10^8^ CFU/mL	24%, 10^8^ CFU/mL
BSFL_3-10_	1,000	400:600	10%, 10^8^ CFU/mL	20%, 10^8^ CFU/mL
BSFL_3-8_	1,000	400:600	8%, 10^8^ CFU/mL	16%, 10^8^ CFU/mL
BSFL_3-6_	1,000	400:600	6%, 10^8^ CFU/mL	12%, 10^8^ CFU/mL
BSFL_4-12_	1,000	200:800	12%, 10^8^ CFU/mL	24%, 10^8^ CFU/mL
BSFL_4-10_	1,000	200:800	10%, 10^8^ CFU/mL	20%, 10^8^ CFU/mL
BSFL_4-8_	1,000	200:800	8%, 10^8^ CFU/mL	16%, 10^8^ CFU/mL
BSFL_4-6_	1,000	200:800	6%, 10^8^ CFU/mL	12%, 10^8^ CFU/mL

### 2.11 Calculations and statistical analysis

Survival rate (Formula 1), development time, larval biomass (total weight of fresh and dried larvae weight per container) (Formula 2), bioconversion rate, feed conversion ratio (FCR) (Formula 3), and waste reduction (Formula 4) were established as processing metrics for BSFL (Rehman et al., 2017a,b, 2019).


(1) $$\begin{array}{l} \mathrm{Survival rate}\;\left(\%\right)=\\ \left[\begin{array}{l} \mathrm{number of larvae}\;\mathrm{at}\;\mathrm{the}\\ \mathrm{terminations of the experiment}/\\ \mathrm{number of larvae}\;\mathrm{at}\;\mathrm{the start of experiment} \end{array}\right]\times 100\% \end{array}$$

(2) $$\begin{array}{l} \mathrm{Larval bioconversion rate}\;\left(\%\right)=\\ \left[\begin{array}{l} \mathrm{dry}\;\mathrm{weight of larvae after conversion}-\\ \mathrm{dry}\;\mathrm{weight of larvae before conversion} \end{array}\right]/\\ \mathrm{dry}\;\mathrm{weight of}\;\mathrm{pig}\;\mathrm{manure before conversion}\times 100\% \end{array}$$

(3) $$FCR\;\left(g/g\right)=\mathrm{feed}\;\mathrm{intake}\;\left(g\right)/\mathrm{gained}\;\mathrm{weight}\;\left(g\right)$$

(4) $$\begin{array}{l} \mathrm{Dry}\;\mathrm{mass reduction}\;\left(\%\right)=\\ \left[\begin{array}{l} \mathrm{mass of feed}\;\mathrm{at}\;\mathrm{the beginning of the experiment}\;\left(g\right)–\\ \mathrm{mass of residue}\;\mathrm{at}\;\mathrm{the terminations of the experiment}\;\left(g\right)/\\ \mathrm{mass of feed}\;\mathrm{at}\;\mathrm{the start of the experiment}\;\left(g\right) \end{array}\right]\\ \;\times 100\% \end{array}$$

SPSS version 19.0 (SPSS, Chicago, Illinois, USA) was used for statistical analysis of the sample’s microbial community diversity and relative richness. In short, all calculations were performed on replicate values and analysis of variance was also performed. The average of three replicates was used for paired t-test analysis. *P* < 0.05 was considered to reflect a statistically significant difference.

## 3 Results

### 3.1 Optimal conversion of Wuzhishan pig manure by BSFL in the different growing stages

This study compared the conversion rates of Black Soldier Fly Larvae (BSFL) from the Wuhan strain when they were raised on pig manure. The study looked at the PW, WP, GP, and SP developmental phases. The results showed significant differences between the groups. Specifically, the GP group performed better than the other groups, with a BSFL survival rate of 96.75%, an individual fresh weight of 0.23 g, an individual dry weight of 0.097 g, and a larval conversion rate of 19.96% (Table 2). Additionally, the GP group showed significant advantages over the other groups in terms of dry matter reduction rate at 43.27% and a feed conversion ratio of 2.17 (Table 3). Furthermore, the study investigated the changes in nutritional elements of Wuzhishan pig manure after converted by BSFL in different growth stages. The lowest total organic carbon (TOC) content in the residue was observed in the GP group (32.65%), while the highest TOC content was observed in the SP group (37.83%). The content of total nitrogen (TN) (1.861%), total phosphorus (TP) (0.2524%), and total potassium (TK) (0.126%) in the substrate in the GP group were significantly lower than that in other groups (Table 4).

**Table 2:** Survival rate, individual fresh and dry larval weight, bioconversion, and larvae weight gain rate (mean ± standard error; *n* = 3).

Treatments	Survival rate (%)	Individual fresh weight (g)	Individual dry weight (g)	Larval bioconversion rate (%)
PW	95.34 ± 1.24^a^	0.217 ± 0.02^a^	0.093 ± 0.01^a^	18.91 ± 0.03^b^
WP	94.52 ± 3.25^a^	0.225 ± 0.03^a^	0.091 ± 0.01^a^	18.90 ± 0.02^b^
GP	96.75 ± 2.53^a^	0.232 ± 0.02^a^	0.097 ± 0.01^a^	19.96 ± 0.01^a^
SP	89.65 ± 1.03^b^	0.189 ± 0.01^b^	0.089 ± 0.01^b^	18.88 ± 0.01^b^
Con	91.28 ± 1.01^a^	0.203 ± 0.01^a^	0.092 ± 0.01^a^	18.92 ± 0.01^b^

Mean values sharing the same letter in a column are not significantly different. ^a^(* *p* < 0.05), ^b^(**p < 0.01).

Pre-weaning (PW), post-weaning piglets (WP), growing pigs (GP), sows (SP), control (con).

**Table 3:** Waste dry matter reduction, feed conversion ratio during black soldier fly larvae rearing on pre-weaning (PW), post-weaning piglets (WP), growing pigs (GP), sows (SP), and control feed (con), (mean ± standard error; *n* = 3).

Treatments	Substrate dry weight (g)	Frass dry weight (g)	Dry matter consumption (g)	Dry matter reduction (%)	Feed conversion ratio
PW	400	241.76 ± 1.26^b^	158.24 ± 2.36^b^	39.56 ± 1.94^b^	1.728 ± 0.24^b^
WP	400	238.48 ± 1.57^a^	161.52 ± 1.35^a^	40.38 ± 1.23^a^	2.025 ± 0.14^a^
GP	400	226.92 ± 2.26^a^	173.08 ± 2.35^a^	43.27 ± 2.02^a^	2.176 ± 0.23^a^
SP	400	249.04 ± 2.55^b^	150.96 ± 3.16^b^	37.74 ± 1.01^b^	1.682 ± 0.33^b^
Con	400	246.68 ± 1.26^b^	153.32 ± 2.35^b^	38.33 ± 2.37^b^	1.572 ± 0.22^b^

Mean values sharing the same letter in a column are not significantly different. ^a^(* *p* < 0.05), ^b^(**p < 0.01).

**Table 4:** Reduction ratio of total organic carbon (TOC), total nitrogen (TN), total phosphorus (TP), total potassium (TK) after the conversion pre-weaning (PW), post-weaning piglets (WP), growing pigs (GP), sows (SP), and control feed (con), (mean ± standard error; *n* = 3).

Group	TOC (%)	TN (%)	TP (%)	TK (%)	Feed conversion ratio
PW	35.24 ± 1.573b	2.128 ± 0.0137b	0.2931 ± 0.0054b	0.1557 ± 0.0062b	1.728 ± 0.24^b^
WP	34.27 ± 1.045a	1.934 ± 0.1491a	0.2769 ± 0.0019a	0.1482 ± 0.0047a	2.025 ± 0.14^a^
GP	32.65 ± 0.874a	1.861 ± 0.0224a	0.2524 ± 0.0028a	0.1268 ± 0.0082a	2.176 ± 0.23^a^
SP	37.83 ± 1.457b	2.235 ± 0.0673b	0.3105 ± 0.0028b	0.1734 ± 0.0055b	1.682 ± 0.33^b^
Con	36.58 ± 2.066b	2.143 ± 0.0245b	0.3034 ± 0.0051b	0.1925 ± 0.0011b	1.572 ± 0.22^b^

Mean values sharing the same letter in a column are not significantly different. ^a^(* *p* < 0.05), ^b^(**p < 0.01).

### 3.2 Correlation between conversion efficiency and gut microbiota of BSFL (Wuhan strain)

**Figure 1:**
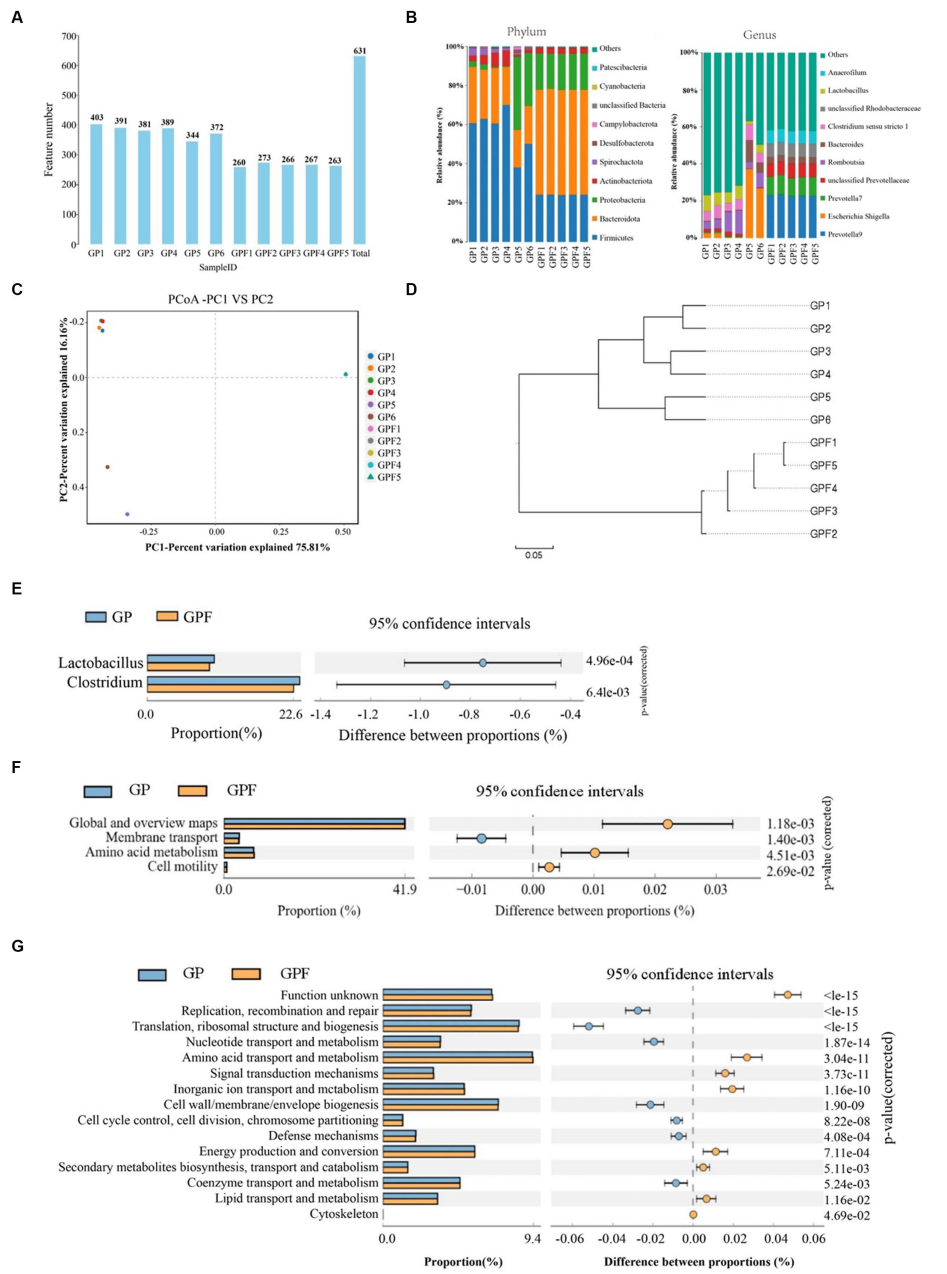
Gut microbiota sequencing analysis of BSFL raised on Wuzhishan pig manure throughout the fattening phase and mature larvae, **(A)** Distribution diagram of the number of OTUs detected, **(B)** Distribution of species in pig manure. **(C)** Principal coordinates analysis (PCoA) in manure microbiota of pigs, **(D)** UPGMA phylogenetic tree analysis, **(E)** Genus’s level differences among samples. **(F)** KEGG enrichment analysis, **(G)** COG functional prediction analysis.

We explore the possible interactions between the gut microbiota and the conversion efficiency of BSFL from the Wuhan strain during the conversion of Wuzhishan pig manure in this section. The gut microbiota of BSFL (Wuhan strain) from two groups: the growth stage pig manure group (GP) and the larvae from the group after the conversion of Wuzhishan pig manure (GPF) were subjected to 16S rRNA sequencing in order to investigate this association. A total of 631 OTUs were detected, with 659,971 valid reads obtained (Figure 1A). Species annotation and taxonomic analysis revealed that among the 11 sampled specimens, a total of 14, 20, 49, 98, 232, and 298 different species were detected at the levels of phylum, class, order, family, genus, and species, respectively. At the phylum level, dominant bacterial phyla in the GP group were Firmicutes, Bacteroidota, Actinobacteriota, and Campylobacterota, while in the GPF group, they were Bacteroidota, Firmicutes, Proteobacteria, and Actinobacteriota. At the genus level, dominant genera in the GP group included *Lactobacillus*, *Clostridium sensu_stricto_1*, *Romboutsia*, and *Escherichia_Shigella*, whereas in the GPF group, they included *Prevotella_9*, *Prevotella_7*, *unclassified_Prevotellaceae*, *unclassified_Rhodobacteraceae*, and *Anaerofilum* (Figure 1B). The principal coordinates analysis (PCoA) showed significant differences in gut microbial structure between the GP group and the control group, with samples within the GP group displaying similarity (Figure 1C). The UPGMA phylogenetic tree analysis demonstrated significant changes in microbial community structure following BSFL treatment of pig manure, with samples within the GP group clustering more closely together, consistent with PCoA results (Figure 1D). To assess differences in microbial community abundance between the two groups, STAMP analysis was performed, revealing that the *Lactobacillus* and *Clostridium* genera were significantly more abundant in the GP group compared to the GPF group (Figure 1E). Additionally, KEGG enrichment analysis identified three significantly different metabolic pathways between GP and GPF: membrane transport, amino acid metabolism, and cell motility metabolism pathways (Figure 1F). Subsequently, COG functional prediction analysis revealed significant differences in 8 annotations between the GP and GPF groups, including replication, recombination, and repair, translation, ribosomal structure, and biogenesis, nucleotide transport, and amino acid transport and metabolism (Figure 1G).

### 3.3 Screening of thermophilic cellulose-degrading bacteria

**Figure 2:**
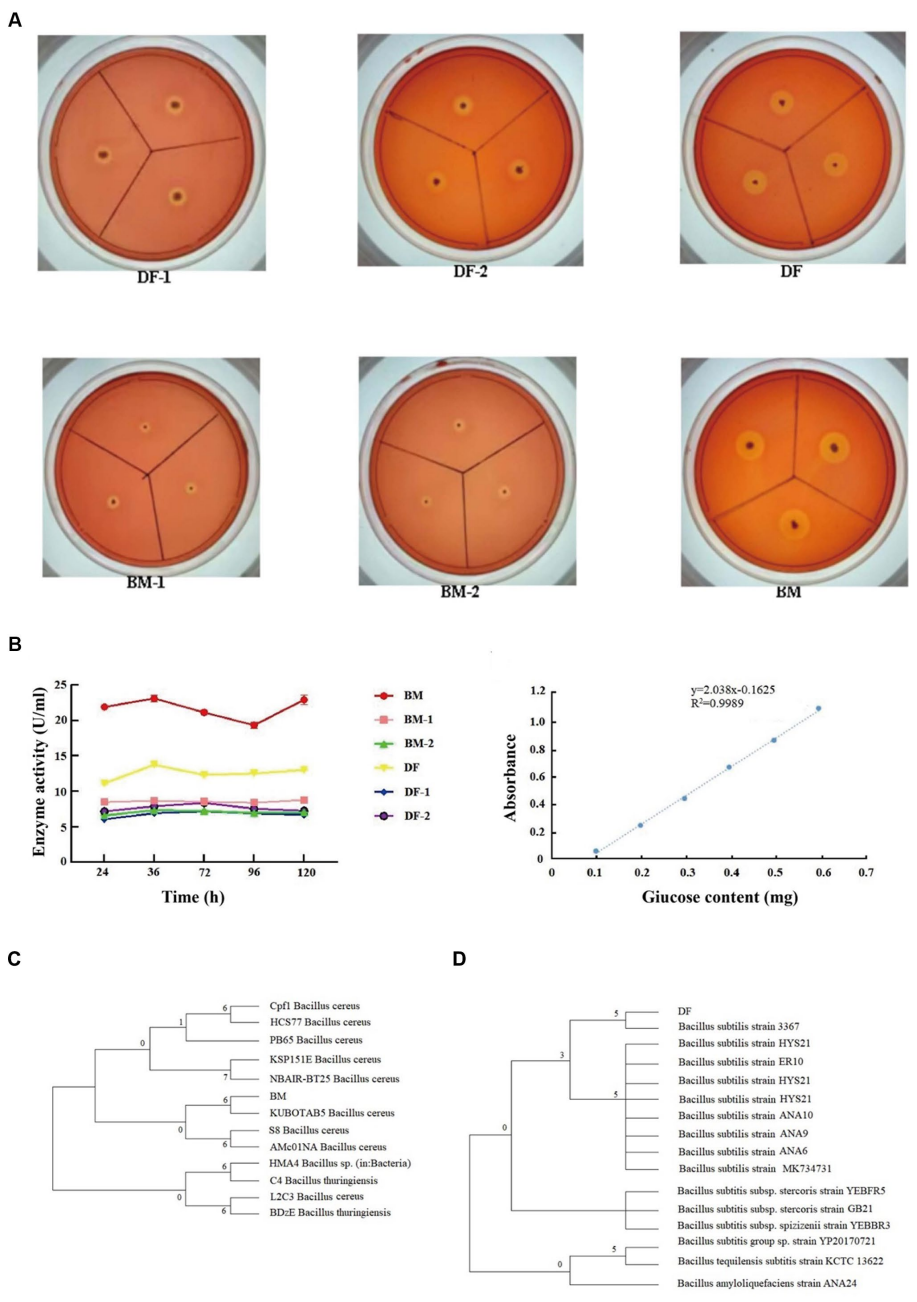
Screening of thermophilic cellulose-degrading bacterial strains. **(A)** Cellulose degradation ability, **(B)** Determination of CMase activity of strains. **(C)** Phylogenetic tree of strain BM. **(D)** Phylogenetic tree of strain DF.

To identify thermophilic cellulose-degrading bacteria present in pig manure and the gut of BSFL, a preliminary screening was conducted based on the size of the cellulose-degrading zone on Congo red agar plates, resulting in the isolation of 6 bacterial strains from 55°C exhibiting strong cellulose-degrading ability. Among them, strains BM and DF exhibited the largest Up values for colony diameter (d) and transparent zone diameter (D), with Up values of 10, indicating the highest cellulose degradation ability within the same group (Figure 2A). Subsequently, the selected strains underwent a secondary screening using CMCase enzyme activity and filter paper degradation assays. The results showed that strain BM had higher CMCase enzyme activity than strain DF, with BM-2 and DF-1 having the lowest CMCase enzyme activity. Strain BM exhibited the highest filter paper degradation rate at 82.64%, followed by strain DF at 81.98%, and strain BM-1 at the lowest rate of 75.31% (Figure 2B). Therefore, strains BM and DF were selected for subsequent strain sequencing and identification. Sequencing results revealed that the full-length 16S rDNA nucleotide sequence of strain BM was 1,423 bp, while that of strain DF was 1,402 bp. Alignment with the NCBI-BLAST database indicated that strain BM was closely related to *Bacillus cereus*, and strain DF was closely related to *Bacillus subtilis*. A phylogenetic tree was constructed using the Neighbor-J method based on the full 16S rDNA sequence, revealing that strain BM belonged to the phylum Firmicutes as *Bacillus cereus* (Figure 2C), and strain DF belonged to the phylum Firmicutes as *Bacillus subtilis* (Figure 2D).

### 3.4 Conversion efficiency with cellulose-degrading bacteria-BSFL synergy

**Figure 3:**
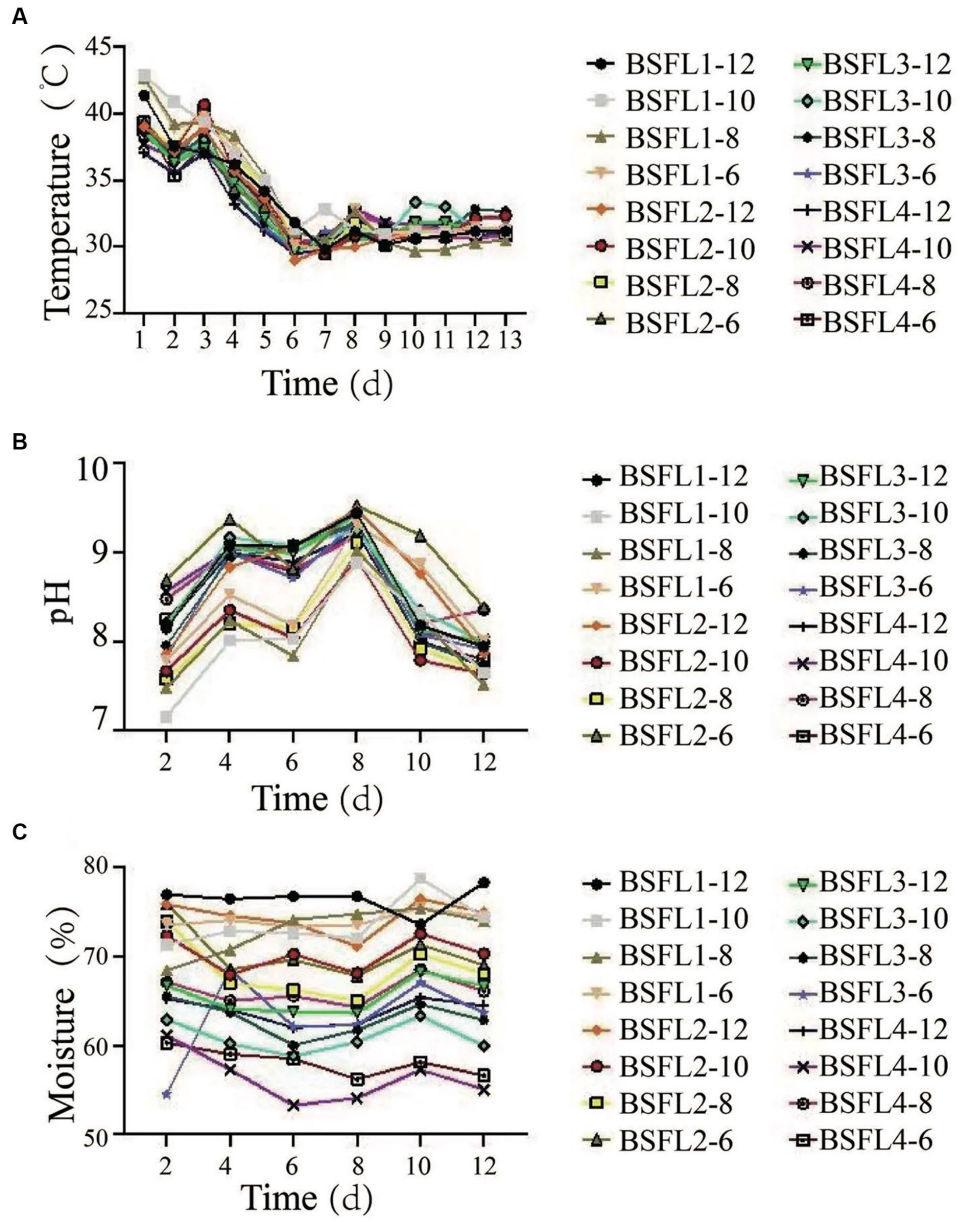
Effects of different temperature, pH value, and water content on co-conversion mixtures ratio of pig manure and pineapple in the conversion process, **(A)** Temperature, **(B)** pH value, **(C)** Water content (%); bars indicate the standard error of the means (*n* = 3).

The selected thermophilic cellulose-degrading bacterial strains and BSFL (Wuhan strain) were co-cultivated in a synergistic manner. Different ratios of Wuzhishan pig manure, pineapple waste, and bacterial strains were mixed to determine the optimal co-cultivation conditions and to assess the conversion efficiency. The results revealed that under the material-to-bacterial strain ratio of the BSFL_1-10_ treatment group, BSFL exhibited optimal growth and biological conversion efficiency. Specifically, the data included larval moisture content (71.11%), residue moisture content (63.20%), larval conversion rate (18.91%), and waste reduction rate (42.28%) (Table 5). The specific temperature, pH and moisture changes during the conversion process are shown in results (Supplementary Figure S1). We monitored temperature changes during the conversion process, and in all groups, temperatures initially decreased, then increased, and finally decreased to reach a stable state with fluctuations. In the BSFL_1-10_ treatment group, the temperature increased to 42.83°C, then decreased to 31.17°C, exhibited slight fluctuations, and eventually stabilized at an equilibrium temperature of 30.08°C (Figure 3A). Furthermore, we tracked pH changes during the conversion process. The BSFL_1-10_ group had the lowest pH value of 7.16, which gradually increased to 7.65, reaching a neutral range, followed by fluctuations around 8.00. The pH values of other groups increased with treatment time, followed by a return to a narrow fluctuation range around neutral, demonstrating an overall trend of an initial increase followed by a decrease, ultimately reaching neutrality (Figure 3B). The initial and final pH values showed minimal differences throughout the treatment process. Slightly alkaline conditions favored organic matter decomposition and microbial activity, promoting the metabolism of BSFL (Wuhan strain). During the entire treatment period, the moisture content of groups BSFL_1-12_, BSFL_1-10_, BSFL_1-8_, and BSFL_1-6_ ranged from 60 to 70% (Figure 3C). Based on the growth observations, it was concluded that a reasonable moisture content of approximately 70% is suitable for the growth environment of BSFL (Wuhan strain). Moisture content below this level could potentially hinder their growth and development or even lead to mortality. The highest conversion efficiency of the mixture of thermophilic cellulose-degrading bacteria BM and DF with BSFL (Wuhan strain) in converting Wuzhishan pig manure and pineapple waste was 18.95%, which showed a significant difference (*p*<0.01) when compared to the control group. The waste reduction rate in the BSFL_1-10_ group was 48.54%, also showing a highly significant difference (*p*<0.01) compared to the control group. Moisture content of mature larvae was found to be in the order of NC_5-10_, NC_6-10_, BSFL_1-10_, and NC_3-10_ groups. The highest residue moisture content was observed in the NC_6-10_ group at 68.94%, while the lowest was in the NC_4-0_ group at 62.06% (Table 6).

**Table 5:** Pineapple crown buds and Wuzhishan pig manure co-conversion efficiency with microbe assisted BSFL technology, (mean ± standard error; *n* = 3).

Treatments	Mature larvae water content (%)	Residue water content (%)	Larval bioconversion rate (%)	Waste reduction rate (%)
BSFL_1-12_	67.41 ± 0.13^a^	59.14 ± 0.35^b^	15.80 ± 0.19^b^	34.46 ± 0.13^b^
BSFL_1-10_	71.11 ± 0.43^a^	63.20 ± 0.42^a^	18.91 ± 0.08^a^	42.28 ± 0.07^a^
BSFL_1-8_	64.18 ± 0.08^b^	50.34 ± 0.28^c^	14.99 ± 0.17^c^	26.46 ± 0.14^d^
BSFL_1-6_	65.78 ± 0.12^b^	62.67 ± 0.66^a^	14.00 ± 0.09^c^	28.91 ± 0.26^d^
BSFL_2-12_	63.25 ± 0.48^b^	60.56 ± 0.22^a^	16.78 ± 0.02^b^	25.85 ± 0.47^d^
BSFL_2-10_	61.30 ± 0.56^c^	62.69 ± 0.26^a^	16.52 ± 0.08^b^	38.12 ± 0.12^b^
BSFL_2-8_	66.43 ± 0.10^b^	45.92 ± 0.36^d^	14.74 ± 0.05^c^	30.17 ± 0.24^b^
BSFL_2-6_	67.34 ± 0.09^a^	57.85 ± 0.25^b^	15.15 ± 0.14^b^	24.71 ± 0.23^d^
BSFL_3-12_	68.09 ± 0.10^a^	53.80 ± 0.38^c^	16.20 ± 0.08^b^	14.16 ± 0.15^e^
BSFL_3-10_	68.05 ± 0.02^a^	49.35 ± 0.12^c^	16.65 ± 0.13^b^	18.48 ± 0.20^e^
BSFL_3-8_	66.52 ± 0.18^b^	53.47 ± 0.33^c^	14.53 ± 0.19^c^	22.59 ± 0.22^d^
BSFL_3-6_	69.05 ± 0.14^a^	44.31 ± 031^d^	15.36 ± 0.05^b^	25.50 ± 0.37^d^
BSFL_4-12_	66.01 ± 0.17^b^	53.10 ± 0.24^c^	15.90 ± 0.18^b^	16.49 ± 0.20^e^
BSFL_4-10_	70.66 ± 0.13^a^	46.53 ± 0.25^d^	14.13 ± 0.15^c^	15.17 ± 0.34^e^
BSFL_4-8_	70.04 ± 0.26^a^	43.20 ± 0.22^d^	15.13 ± 0.08^b^	15.98 ± 0.24^e^
BSFL_4-6_	64.56 ± 0.11^b^	48.21 ± 0.10^c^	14.13 ± 0.11^c^	17.97 ± 0.19^e^

Mean values sharing the same letter in a column are not significantly different. ^a^(* *p* < 0.05), ^b^(**p < 0.01).

**Table 6:** Investigation of BSFL ratio (pig manure and pineapple), (mean ± standard error; *n* = 3).

Treatments	Final larval moisture content (%)	Residue moisture content (%)	Larval bioconversion rate (%)	Waste reduction rate (%)
BSFL_1-10_	71.66 ± 1.11b	66.70 ± 1.20a	18.95 ± 0.32a	48.54 ± 1.65a
NC_2-10_	-	64.22 ± 0.92b	-	-
NC_3-10_	71.40 ± 1.66b	67.13 ± 1.14a	12.44 ± 0.67b	36.20 ± 1.81b
NC_4-0_	-	62.06 ± 1.52b	-	-
NC_5-10_	76.99 ± 0.21a	68.49 ± 1.46a	14.36 ± 0.19b	31.95 ± 1.03b
NC_6-10_	75.34 ± 0.43a	68.94 ± 1.57a	11.54 ± 0.47b	32.84 ± 1.68b

Mean values sharing the same letter in a column are not significantly different (*p* < 0.05).

BSFL_1-10_: Mixture of pineapple waste and pig manure with added bacteria and larvae; NC_2-10_: Mixture of pineapple waste and pig manure with added bacteria but no larvae; NC_3-10_: Mixture of pineapple waste and pig manure with no bacteria but added larvae; NC_4-0_: Mixture of pineapple waste and pig manure with no bacteria and no larvae; NC_5-10_: Pineapple waste with no bacteria and no larvae; NC_6-10_: Pig manure with no bacteria and no larvae.

## 4 Discussion

The study presents intriguing findings regarding the best conversion of Wuzhishan pig manure by BSFL at various growth stages, the relationship between conversion efficiency and BSFL gut microbiota, the screening of thermophilic cellulose-degrading bacteria, and the efficiency of conversion attained through the synergy between cellulose-degrading bacteria and BSFL. The findings suggest possible directions for increasing the effectiveness of this method and give insight into a variety of features of insect-assisted garbage treatment.

The results showed GP group performed better than the other groups, with a BSFL survival rate of 96.75%, an individual fresh weight of 0.23 g, an individual dry weight of 0.097 g, and a larval conversion rate of 19.96% (Table 2). Additionally, the GP group showed significant advantages over the other groups in terms of dry matter reduction rate at 43.27% and a feed conversion ratio of 2.17 (Table 3). The former investigation in line with the present investigation in terms of survival rate, larvae weight gain, dry matter content of the larvae, and waste reduction rate during fresh animal manure valorization with BSFL (Oonincx et al., 2015; Rehman et al., 2017a,b; Veldkamp et al., 2021). Notably, the residue’s TOC content was lowest in the GP group, suggesting that the group’s larvae were successful in metabolizing and lowering the organic carbon content of the substrate. This finding supports the GP group’s enhanced dry matter reduction rate and highlights the method’s potential for managing organic waste. Further evidence supporting the involvement of BSFL in changing the nutritional content of the waste substrate comes from the changed amounts of TN, TP, and to TK in the GP group. The TOC, TN, TP reduction rate during the BSFL development on dairy manure, chicken manure and soybean curd residues was first time reported (Rehman et al., 2017a,b), as per best of our knowledge, further it was later reported in many investigations (Barbi et al., 2022; Naser El Deen et al., 2023). The large decrease in these components in the GP group as compared to other groups implies that the larvae are crucial to the conversion and cycling of nutrients during waste processing.

This study investigated the relationship between the gut microbiota and the BSFL ability to convert Wuzhishan pig manure. Taxonomic study showed that the GP and GPF groups had different microbial compositions, with Firmicutes, Bacteroidota, Actinobacteriota, and Campylobacterota predominating in GP and Bacteroidota, Firmicutes, Proteobacteria, and Actinobacteriota predominating in GPF. The microbial communities of BSFL varies as per substrate used for the larval rearing and stages of larvae development (De Smet et al., 2018; Schreven et al., 2022; Auger et al., 2023). The species difference analysis using STAMP revealed that the abundance of the *Lactobacillus* and *Clostridium* in the GP group was significantly higher than in the GPF group, indicating that pig manure at different developmental stages can alter the structure of the BSFL gut microbiota. Moreover, Principal Coordinates Analysis (PCoA) revealed significant variations, with coherence in GP samples. According to an abundance study, GP had greater levels of *Lactobacillus* and *Clostridium*, which may have contributed to the effective conversion of pig manure. The association between microbial shifts and metabolic alterations is demonstrated by the identification of many metabolic pathways by KEGG analysis, such as membrane transport, amino acid metabolism, and cell motility metabolism pathways (Yu et al., 2022; Kariuki et al., 2023). Significant variations in annotations were found by COG analysis, pointing to BSFL-driven changes in the structure and functioning of microbial communities (Auger et al., 2023; Pei et al., 2023). These results contribute to our understanding of waste conversion based on BSFL and its potential to improve resource recovery strategy. But it should be noted that PICRUSt2 as a prediction tool, has its limitations, as biases may arise from the reference databases, and its resolution cannot distinguish strain-specific functions. Therefore, these predictive findings must be considered exploratory and should be interpreted with caution (Douglas et al., 2020; Ou et al., 2022).

Cellulose, a primary component in pig manure in the composting process. The efficient degradation of cellulose in pig manure is pivotal for the success of fermentation (Zeng et al., 2022). Cellulose-degrading bacteria secrete cellulases that effectively break down cellulose in pig manure. Therefore, the selection of thermophilic cellulose-degrading bacteria under high-temperature conditions and their application in collaboration with BSFL can mitigate the impact of high temperatures on biodiversity during composting. This represents an effective approach to improve pig manure composting techniques and optimize cellulose utilization. In our former research, the manure microbiota of Wuzhishan pigs at different growth stages was investigated using high throughput 16S rRNA sequencing. Firmicutes and Bacteroidetes were the two dominant phyla in four stages: including pre-weaning (PW), post-weaning piglets (WP), growing pigs (GP), and sows (SP). the proportion of Firmicutes increased in GP and SP stages (Shao et al., 2021). Firmicutes were the most dominant bacterial phylum with an abundance of 59% in larvae fed with pig manure and 74% in larvae fed with dairy manure (Zhan et al., 2020). We examined the BSFL gut and pig manure for microorganisms that break down thermophilic cellulose. Six bacteria were discovered during the initial Congo red agar screening that had strong cellulose breakdown at 55°C. Notably, strains BM and DF outperformed others in the same group, displaying the largest colony and transparent zone diameters (Up values of 10) (Figure 2A). Additionally, strain BM showed higher CMCase enzyme activity than strain DF, and strain BM also showed the greatest rate of filter paper degradation, at 82.64%, followed by strain DF at 81.98% (Figure 2B). Sequencing revealed that BM was *Bacillus cereus* and DF was *Bacillus subtilis*, which led to the decision to characterize them. The 16S rDNA sequences of strains BM and DF, which aligned with the Firmicutes phylum, were 1,423 bp and 1,402 bp, respectively (Figures 2C,D). The bacteria BM and DF were identified through this screening as promising cellulose degraders with uses in biotechnology and waste management. The results are in agreement with former investigation where the enzyme producing microbial communities from the BSFL and manure was identified (De Smet et al., 2018; Rehman et al., 2019; Gorrens et al., 2021).

Pig manure contains abundant proteins, fats, and cellulose, as well as a substantial amount of organic matter, nitrogen, phosphorus, and potassium elements (Meng et al., 2018). Pineapple waste is rich in vitamins, minerals, and dietary fiber. The conversion of pineapple waste and pig manure by BSFL from the Wuhan strain contributes to environmental protection and resource recycling. Some research showed that *Bacillus* is in high abundance in the BSFL intestine. *Bacillus* can degrade lignocellulose (De Smet et al., 2018; Zhan et al., 2020). But there is no research in screening thermophilic cellulose-degrading strains from Wuzhishan pig manure and gut of BSFL that converted Wuzhishan pig manure and pineapple crown buds by-production.

To maximize conversion efficiency, a number of thermophilic cellulose-degrading bacterial strains were co-cultivated with the Wuhan strain of BSFL in a synergistic manner. We evaluated the efficacy of the co-cultivation by adjusting the ratios of pig manure, pineapple waste, and bacterial strains. The BSFL_1-10_ treatment group produced the best growth and biological conversion efficiency, according to the results. Larval moisture content (71.11%), residual moisture content (63.20%), larval conversion rate (18.91%), and waste reduction rate (42.28%) measures were used to demonstrate this (Table 5). Temperature changes were seen during the conversion process, with the BSFL_1-10_ group showing a stabilizing tendency around 30.08°C following early swings (Figure 3A). In the BSFL_1–10_ group, pH monitoring showed the lowest pH value of 7.16, which steadily increased to neutrality with minor oscillations around 8.00 (Figure 3B). According to growth observations, keeping the moisture level at roughly 70% was best for the development of the BSFL (Wuhan strain) (Figure 3C). Surprisingly, co-cultivation with the thermophilic cellulose-degrading bacteria BM and DF increased conversion efficiency significantly, with the mixture obtaining an astounding 18.95% conversion efficiency and a waste reduction rate of 48.54% in the BSFL_1-10_ group (Table 6). This work adds to our knowledge of sustainable waste management techniques by offering useful insights into optimizing waste conversion through co-cultivation tactics. The results of our investigation are in accordance with the former investigation in which gut bacterial strains and exogenous cellulose degrading bacteria was utilized for assistance of BSFL and it was examined the conversion efficiency of cellulose rich organic wastes enhanced during the larvae rearing (Rehman et al., 2019; Somroo et al., 2019; Mazza et al., 2020).

## 5 Conclusion

In conclusion, this study provides an extensive investigation into the transformative role of Black Soldier Fly Larvae (BSFL) in the conversion of Wuzhishan pig manure and pineapple waste. The findings unequivocally underscore the growth stage pig manure group (GP) as the most proficient in enhancing BSFL performance, exhibiting superior survival rates, larval conversion rates, and dry matter reduction rates compared to other groups. Moreover, the exploration of the intricate relationship between conversion efficiency and the gut microbiota of BSFL Wuhan strain reveals significant microbial dynamics that reinforce effective conversion processes. Furthermore, the study meticulously identifies and evaluateds thermophilic cellulose-degrading bacterial strains within the BSFL gut and pig manure. Notably, two promising strains, BM (*Bacillus cereus*) and DF (*Bacillus subtilis*), are identified for their exceptional cellulose-degrading capabilities. The synergistic co-cultivation of these strains with BSFL demonstrates a remarkable surge in conversion efficiency, showcasing the potential of such symbiotic alliances in expediting waste degradation and facilitating nutrient recovery. Additionally, the study delves into the molecular and biochemical pathways associated with cellulose degradation within the collaborative synergy of BSFL and bacteria, presenting a promising approach for the development of innovative enzymatic solutions in organic waste treatment, specifically targeting pig manure and pineapple waste. Although our findings present encouraging insights into refining waste conversion processes using BSFL and thermophilic cellulose-degrading bacteria, it is crucial to acknowledge the inherent limitations of this study. Further research endeavors are warranted to comprehensively elucidate the intricate mechanisms underpinning the relationships between gut microbiota and conversion efficiency. Nevertheless, this work establishess a robust foundation for future investigations aimed at advancing sustainable waste management, driveing resource recovery initiatives, and championing environmental conservation.

## Data availability statement

The datasets presented in this study can be found in online repositories. The names of the repository/repositories and accession number(s) can be found in the article/Supplementary material.

## Author contributions

MS: Data curation, Formal analysis, Investigation, Methodology, Writing – original draft, Writing – review & editing. XZ: Data curation, Formal analysis, Writing – review & editing. KR: Writing – review & editing. MC: Writing – original draft. LZ: Writing – original draft. FH: Writing – review & editing. JZ: Funding acquisition, Project administration, Writing – review & editing.
