# Supplementary figures and images for: Synergistic bioconversion of organic waste by black soldier fly (Hermetia illucens) larvae and thermophilic cellulose-degrading bacteria
Source: Front Microbiol. 2024 Jan 10;14:1288227. doi: 10.3389/fmicb.2023.1288227 (PMC10806183; doi:10.3389/fmicb.2023.1288227)

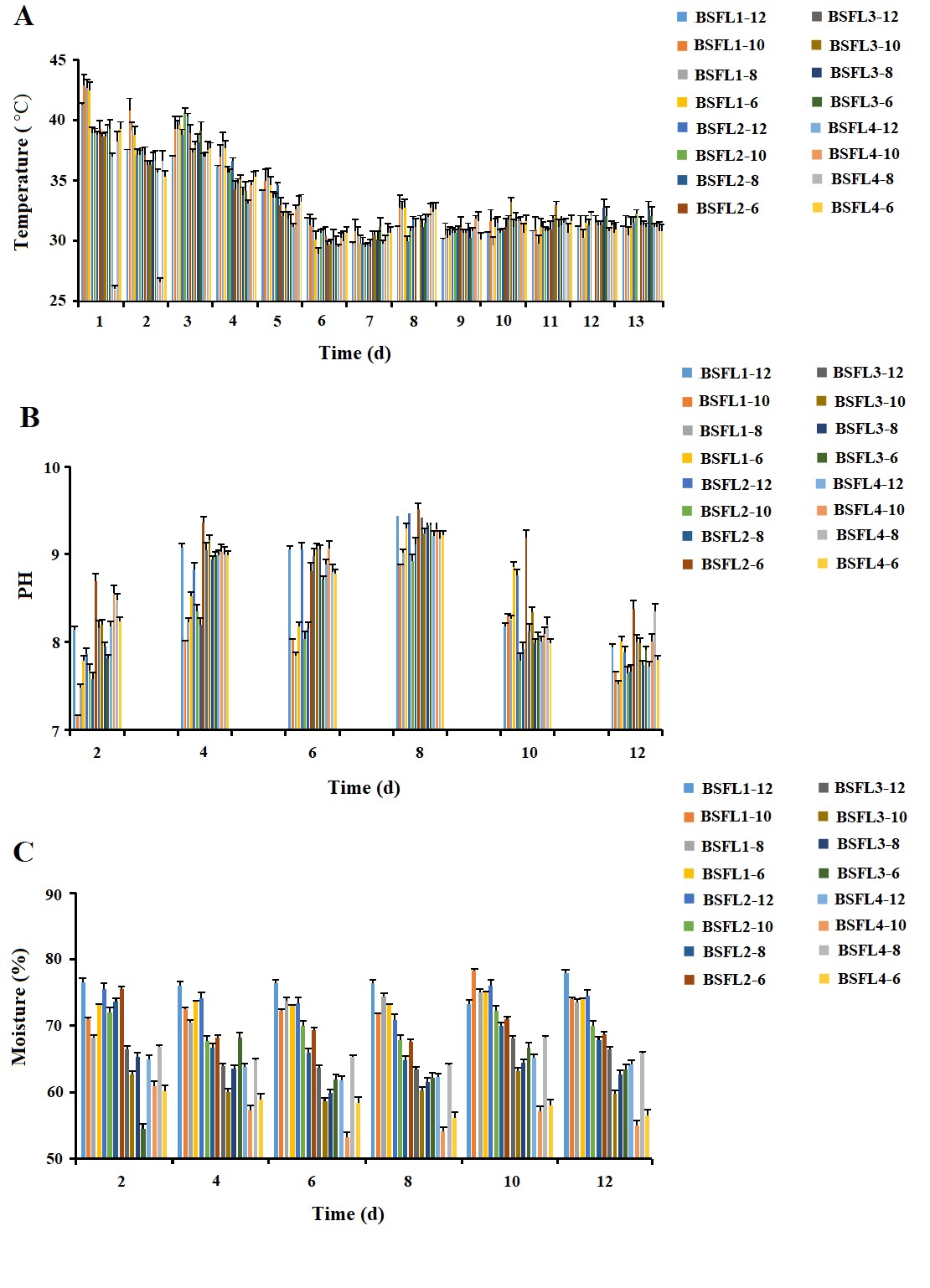

Supplement: Supplementary file 1 [file Image_1.JPEG]
